# A possible role of the locus coeruleus in complex regional pain syndrome

**DOI:** 10.3389/fnint.2012.00104

**Published:** 2012-11-08

**Authors:** Peter D. Drummond

**Affiliations:** School of Psychology, Murdoch UniversityPerth, WA, Australia

**Keywords:** complex regional pain syndrome, hemilateral pain modulation, locus coeruleus, startle, psychological distress

## Abstract

Heightened sensitivity to painful stimulation commonly spreads from the affected limb to the ipsilateral forehead in patients with complex regional pain syndrome (CRPS). In addition, acoustic startle evokes greater auditory discomfort and increases in limb pain when presented on the affected than unaffected side. In contrast, limb pain ordinarily evokes analgesia in the ipsilateral forehead of healthy participants, and acoustic startle suppresses limb pain. Together, these findings suggest that hemilateral and generalized pain control mechanisms are disrupted in CRPS, and that multisensory integrative processes are compromised. Failure to inhibit nociceptive input from the CRPS-affected limb could sensitize spinal and supraspinal neurons that receive convergent nociceptive and auditory information from hemilateral body sites. Somatosensory, auditory, and emotional inputs may then aggravate pain by feeding into this sensitized nociceptive network. In particular, a disturbance in hemilateral pain processing that involves the locus coeruleus could exacerbate the symptoms of CRPS in some patients.

## Introduction

Complex regional pain syndrome (CRPS) usually begins after a fracture, contusion or sprain, but can develop after a minor injury that would normally heal quickly. Patients typically describe a burning sensation that is aggravated by movement, the limb being touched, and ambient temperature changes. The pain and sensory disturbances are often so severe that they lead to profound disability (Marinus et al., 2011).

In the first detailed description of this syndrome, Mitchell (1872) noted that arousal stimuli and emotional distress greatly intensified pain. We recently confirmed this in controlled studies that involved a standard auditory startle stimulus. Patients reported that startle stimuli presented on the CRPS-affected side sounded louder and provoked greater increases in limb pain than contralateral startle stimuli (Knudsen et al., 2011). In contrast, heat-pain *decreased* momentarily in healthy participants when they were startled (Drummond et al., 2001).

We also found that sensitivity to painful mechanical pressure generally extended from the CRPS-affected limb to the ipsilateral forehead (Drummond and Finch, 2006; Finch et al., 2009), whereas pain referral to the ipsilateral forehead was uncommon in other forms of chronic unilateral pain (Knudsen et al., 2011). Since the locus coeruleus responds to nociceptive and alerting stimuli (Aston-Jones et al., 1991; Jones, 1991; Van Bockstaele et al., 2001; Valentino and Van Bockstaele, 2008), and is implicated in hemilateral pain modulation (Tsuruoka et al., 2004), these observations suggest an involvement of the locus coeruleus in CRPS.

## Bidirectional influences of spinal noradrenergic activity on pain

In emergencies, alarm signals emanating from cortical fear networks inhibit pain messages as they enter the spinal cord, akin to closing a “pain gate.” This inhibitory response, known as “stress-induced analgesia”, is mediated, in part, by spinal release of noradrenaline (Millan, 2002). Specifically, the central noradrenergic system (which alerts the brain to the presence of threats and rewards) activates α_2_-adrenoceptors in the dorsal horn. In turn, this inhibits the release of excitatory neurotransmitters from primary nociceptive afferents and blocks pain. The effectiveness of this anti-nociceptive mechanism sometimes strengthens following peripheral nerve injury (Ma and Eisenach, 2003; Hayashida et al., 2012). However, under certain conditions, this spinal anti-nociceptive influence may be compromised after peripheral nerve injury (Rahman et al., 2008), thereby contributing to pain.

In addition to inhibitory influences mediated by α_2_-adrenoceptors, noradrenaline can activate an *excitatory* subclass of adrenergic receptors on primary nociceptive afferents (the α_1_-adrenoceptors) (Millan, 1999). Not only does this intensify pain and hyperalgesia (heightened sensitivity to painful stimulation) but it also evokes axon reflexes (a fundamental component of neurogenic inflammation) (Ren et al., 2005; Drummond, 2009, 2011). Consequently, during acute inflammation or after nerve injury, noradrenergic *facilitation* of pain and inflammation may replace inhibitory noradrenergic effects (Ali et al., 1999; Dogrul et al., 2006; Donello et al., 2011).

Likewise, noradrenaline may sometimes facilitate central nociceptive activity (Millan, 2002). For example, in a model of inflammatory pain, excitation of α_1_-adrenoceptors in the dorsal horn of rats opposed an anti-nociceptive effect mediated by α_2_-adrenoceptors (Jeong and Holden, 2009). This study involved stimulation of the lateral hypothalamus with microinjection of carbachol, thereby activating adrenergic brainstem neurons. Under these conditions, inthrathecal injection of the α_1_-adrenoceptor antagonist WB4101 increased the foot withdrawal latency to noxious heating of the inflamed ankle, indicating that spinal α_1_-adrenoceptors exerted a pro-nociceptive effect. Similar effects were obtained when brainstem adrenergic neurons were activated indirectly with pontine microinjection of morphine (Holden et al., 1999) or the GABA_A_ antagonist bicuculline (Nuseir and Proudfit, 2000). Activation of brainstem α_1_-adrenoceptors may also suppress pain-inhibitory processes, thereby facilitating pain. For example, microinjection of the α_1_-adrenoceptor agonist phenylephrine close to the nucleus raphe magnus suppressed diffuse noxious inhibitory controls in rats (Makino et al., 2010), consistent with a disruptive role of α_1_-adrenoceptors on this pain modulation process.

Additional convergent evidence suggests that brainstem adrenergic nuclei are involved in pain-facilitation. For example, following intraplantar formalin injection, locus coeruleus-lesioned rats spent less time licking or lifting the inflamed hindpaw during a hotplate test than control rats (Martin et al., 1999; Taylor et al., 2000). In a related study, signs of neural activity increased in the locus coeruleus following spared sural nerve injury, and the increase correlated with the intensity of tactile allodynia (pain to a normally innocuous stimulus) (Brightwell and Taylor, 2009). Importantly, targeted destruction of noradrenergic neurons in the locus coeruleus by intra-cerebro-ventricular injection of anti-dopamine-β-hydroxylase-saporin (DβH-saporin) prevented the development of allodynia and hyperalgesia, as did microinjection of local anesthetic agent directly into the locus coeruleus. Similarly, following spared sural nerve injury, microinjection of phenylephrine into the brainstem dorsal reticular nucleus induced hyperalgesia and allodynia; conversely, a reduction in synthesis of noradrenaline in neurons that projected to this nucleus attenuated signs of pain (Martins et al., 2010). In contrast to these reports, spinal depletion of noradrenergic neurons after inthrathecal administration of DβH-saporin *enhanced* signs of mechanical hyperalgesia associated with spinal nerve ligation (Jasmin et al., 2003; Hayashida et al., 2012). Together, these findings suggest that complete destruction of spinal noradrenergic pathways (with partial destruction of brainstem noradrenergic nuclei) augments pain (Jasmin et al., 2003; Hayashida et al., 2012), whereas complete destruction of the locus coeruleus (but sparing most noradrenergic neurons in the A5 and A7 brainstem nuclei) *inhibits* pain (Brightwell and Taylor, 2009). Thus, after peripheral nerve injury, a spinal noradrenergic anti-nociceptive mechanism that projects from the locus coeruleus could transform into a pro-nociceptive mechanism, possibly involving ascending noradrenergic projections in addition to descending coeruleospinal pathways.

In an important study by Hodge et al. (1983), the effect of locus coeruleus stimulation on the firing rate of cells in lamina 4–5 of the dorsal horn to noxious skin stimulation was studied after dorsal root rhizotomy. On the side contralateral to the rhizotomy, locus coeruleus stimulation inhibited neural responses to noxious skin stimulation. However, on the lesioned side, locus coeruleus stimulation sometimes *facilitated* responses in dorsal horn neurons, consistent with the transformation of a normal inhibitory coeruleospinal influence into a facilitatory effect. In addition to spinal nociceptive projections, a separate population of noradrenergic neurons in the locus coeruleus supplies the somatosensory thalamus (Voisin et al., 2005) where they exert excitatory and inhibitory effects on nociceptive traffic via α_1_- and α_2_-adrenoceptors, respectively (Zhang et al., 1998). Hence, under certain conditions, activation of the central noradrenergic system might facilitate nociceptive neurotransmission, thereby sensitizing nociceptive neurons at multiple ascending levels.

## Hemilateral pain modulation in an animal model of acute inflammatory pain

In rats, coeruleospinal pain modulation appears to be active in the dorsal horn ipsilateral, but not contralateral, to the site of paw inflammation (Tsuruoka et al., 1999, 2003, 2004). Moreover, the inhibitory influence extends beyond the involved segment to distant ipsilateral segments. For example, 4 h after carrageenan-induced hindpaw inflammation, heat hyperalgesia was detected not only in the inflamed hindpaw but also in the ipsilateral non-inflamed forepaw (Tsuruoka et al., 2004). The hyperalgesia intensified both in the inflamed hindpaw and the ipsilateral forepaw of animals with bilateral lesions of the locus coeruleus, but did not develop in the contralateral hind- or forepaw. When microdialysis was used to measure the concentration of noradrenaline in the ipsilateral dorsal horn, noradrenaline increased within 1 h of the carrageenan injection (Tsuruoka et al., 1999). Importantly, however, levels did not change in the dorsal horn contralateral to inflammation, and remained stable during inflammation in the ipsilateral dorsal horn of rats with bilateral lesions of the locus coeruleus. The effect of a unilateral or bilateral lesion of the locus coeruleus-subcoeruleus on paw withdrawal latencies to noxious heat was investigated by Maeda et al. (2009). Four hours after carrageenan injection into the hindpaw, paw withdrawal latencies were shorter in animals with bilateral lesions than in animals with unilateral lesions and in sham-operated controls, consistent with an inhibitory influence emanating from the locus coeruleus. However, hyperalgesia was equivalent in sham-operated controls and in animals with a unilateral lesion on either side. Together, these findings suggest that carrageenan inflammation activates adrenergic neurons that project from both sides of the locus coeruleus to the ipsilateral dorsal horn, and that activity on only one side is sufficient to inhibit pain.

**Figure 1:**
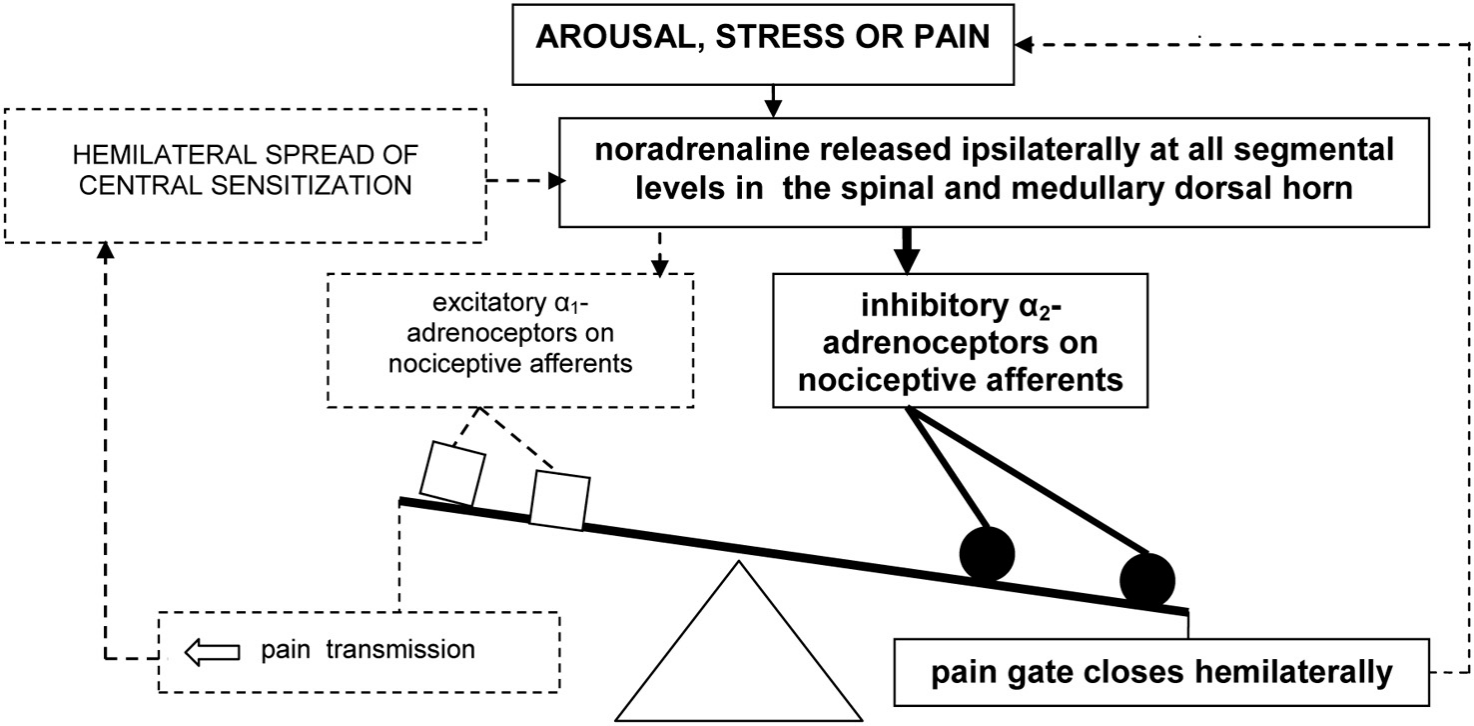
**Model of hemilateral *inhibitory* pain modulation (in bold).** The dashed lines represent failure to engage hemilateral *facilitatory* pain transmission and associated emotional distress.

In sum, pain may evoke the ipsilateral release of noradrenaline in the medullary and dorsal horn. Speculatively, arousal and stress associated with pain could further increase this release. Under normal conditions, a hemilateral inhibitory influence of noradrenaline, mediated by α_2_-adrenoceptors, then closes the spinal “pain gate” (Figure 1).

## Hemilateral pain modulation in healthy humans

We recently identified the counterpart of this hemilateral anti-nociceptive response in healthy humans. In our first study, sensitivity to sharp and blunt pressure was measured on each side of the forehead before and after immersion of one hand in painfully cold water (Knudsen and Drummond, 2009). Immersion of the hand in mildly painful (10°C) water was ineffective, whereas sensitivity to blunt pressure decreased on both sides of the forehead following a single immersion of the hand in water at 2°C or repeated immersions at 4°C (intensely painful). Importantly, this decrease was greater on the ipsilateral than contralateral side of the forehead, consistent with hemilateral pain modulation.

These findings were at odds with animal studies that demonstrated hyperalgesia in the ipsilateral forepaw after hindpaw injection of the inflammatory agent carrageenan (Tsuruoka et al., 2004). Therefore, in a second experiment, sensitivity to mechanical stimulation was assessed on each side of the forehead during two days of treatment of the forearm with topical capsaicin, a substance that induces neurogenic inflammation (Knudsen and Drummond, 2011). Again, the decrease in sensitivity to blunt pressure was greater on the ipsilateral than contralateral side of the forehead when the forearm site was heated. Thus, limb pain, possibly compounded by emotional distress, appears to provoke analgesia in the ipsilateral forehead of healthy humans.

## Up-regulation of α_1_-adrenoceptors on nociceptive afferent fibers after nerve injury

It has long been suspected that α_1_-adrenoceptors are expressed on primary nociceptive afferents. Messenger RNA for α_1_-adrenoceptors is present in the dorsal root ganglia (DRG) of rats (Nicholson et al., 2005), and noradrenaline and the α_1_-adrenoceptor agonist phenylephrine increase the excitability of cultured DRG neurons (Kasai and Mizumura, 2001; Pluteanu et al., 2002). We recently used immunohistochemistry to confirm the expression of α_1_-adrenoceptors within sub-populations of nociceptive neurons in the DRG and skin of rats (Dawson et al., 2011). These neurons expressed α_1_-adrenoceptors, thus providing a histochemical substrate for direct excitation by adrenergic agonists (Gibbs et al., 2008).

Injury to adrenergic nerve fibers within the central nervous system triggers signs of denervation supersensitivity, mediated by an increased density of post-synaptic adrenergic receptors (Roudet et al., 1993; Giroux et al., 1999). Likewise, within the peripheral nervous system, nerve injury provokes signs of an up-regulated expression of adrenergic receptors. For example, the proportion of DRG neurons that responded to noradrenaline increased markedly in rats after chronic nerve injury induced by loose or tight ligation of the sciatic nerve (Petersen et al., 1996), and systemic injection of the α_1_-adrenoceptor antagonist prazosin inhibited nociceptive fiber discharge in other neuropathic pain models (Nam et al., 2000; Hord et al., 2001; Kim et al., 2005). Moreover, messenger RNA for α_1B_-adrenoceptors increased in the DRG following peripheral nerve section or ligation of spinal nerves supplying those ganglia (Xie et al., 2001; Maruo et al., 2006).

Similar effects have been observed in additional models of peripheral neuropathy. For example, stimulation of α_1_-adrenoceptors aggravated allodynia in an animal model of painful diabetic neuropathy; moreover, messenger RNA and binding sites for α_1_-adrenoceptors increased in the DRG (Lee et al., 2000). In addition, cells in rat DRG infected with the varicella-zoster virus gained an unusual sensitivity to the α_1_-adrenoceptor agonist phenylephrine (Schmidt et al., 2003). Together, these findings suggest that heightened α_1_-adrenoceptor activity may aggravate symptoms in various neuropathic pain syndromes.

## Hypothesis: up-regulated α_1_-adrenoceptors disrupt hemilateral pain modulation in CRPS

During the early stages of CRPS, affected skin typically is flushed, hot and dry, implying diminished sympathetic vasoconstrictor and sudomotor activity; but later on the skin becomes cold, clammy and cyanotic, suggestive of excessive sympathetic neural discharge (Birklein, 2005). Despite this clinical picture, there is no direct evidence of an increase in sympathetic outflow in chronically-affected limbs (Casale and Elam, 1992; Dotson, 1993), and no increase in reflex vasoconstrictor responses (Wasner, 2010). In fact, levels of venous noradrenaline and its metabolites are *lower* in the affected than unaffected limbs of patients with CRPS (Drummond et al., 1991; Harden et al., 1994). Nevertheless, directly applied adrenergic agents provoke augmented vasoconstrictor responses in the CRPS-affected limb (Arnold et al., 1993) and in animal models of CRPS (Kurvers et al., 1998; Xanthos et al., 2008). One explanation for this paradox is that an underlying sympathetic deficit, possibly triggered by the inciting trauma, initiates compensatory increases in the sensitivity or expression of adrenergic receptors in affected tissues.

**Figure 2:**
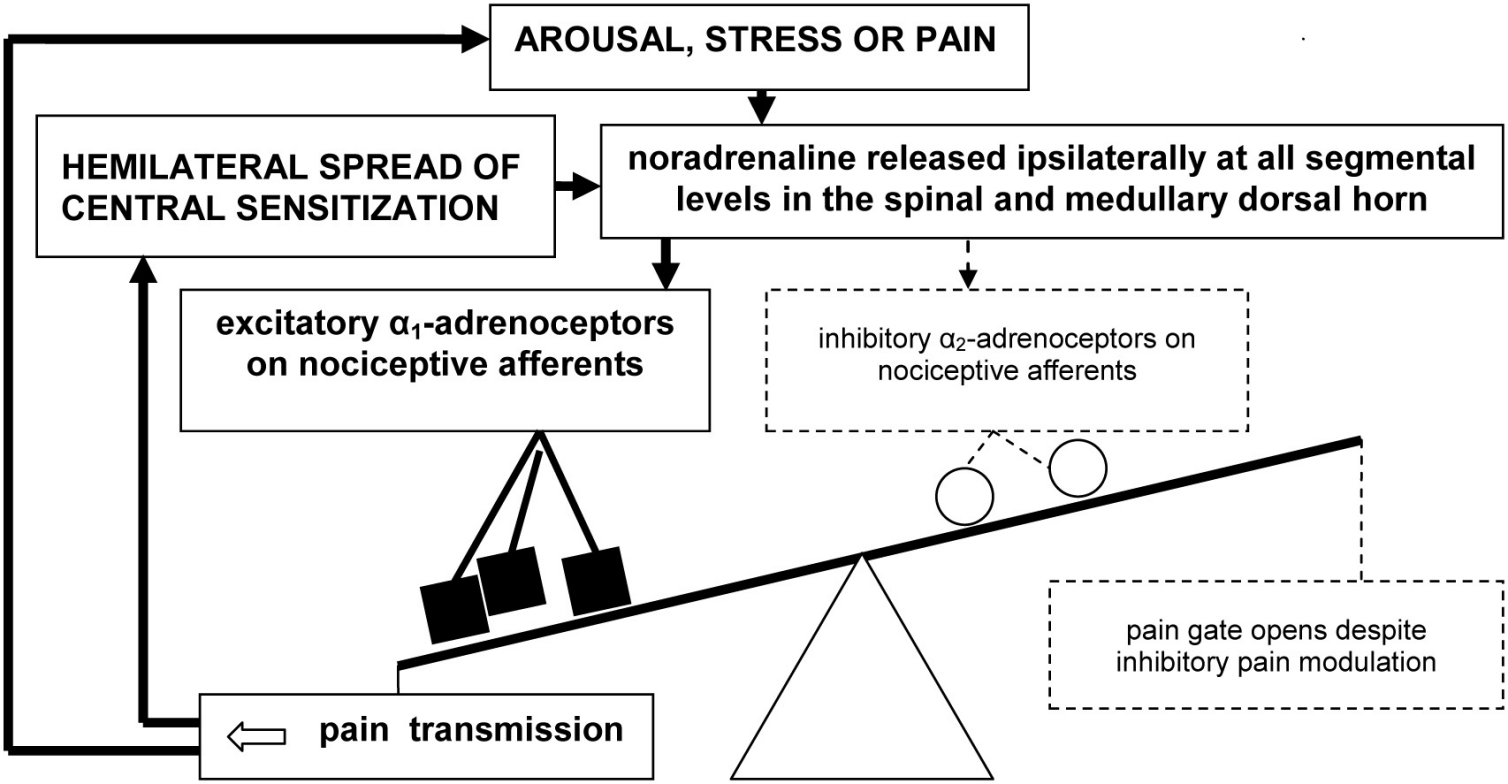
**A vicious cycle involving up-regulated α_1_-adrenoceptors on primary nociceptive afferents may overwhelm inhibitory spinal pain modulation in CRPS and certain other chronic neuropathic pain syndromes, resulting in pain escalation and emotional distress**.

In our working model of pain mediation in CRPS (Drummond, 2010), we propose that the excitatory actions of α_1_-adrenoceptors overwhelm the inhibitory actions of α_2_-adrenoceptors both within peripheral and central nociceptive pathways (Figure 2). Specifically, we hypothesize that the nociceptive effects of noradrenaline, mediated by α_1_-adrenoceptors, effectively mask opposing inhibitory influences of noradrenaline, mediated by α_2_-adrenoceptors.

The excitatory effects of noradrenaline may trigger the hemilateral spread of sensitization from activated second-order nociceptive neurons in the dorsal horn to higher-order convergent neurons in the brainstem, thalamus, and cerebral cortex. In turn, amplified pain messages could switch on pain modulation processes that attempt to close the pain gate. Consequently, activation of the central noradrenergic system during heightened states of arousal, psychological stress—and possibly even pain itself—may paradoxically intensify pain. If so, a spiral of noradrenaline release and pain escalation may prevent normal pain resolution. This new concept could be important not only for clarifying mechanisms of symptom expression in neuropathic pain disorders such as CRPS, but also for identifying new treatment targets.

## Hypothesized mechanism of symptom expression in CRPS

A pro-nociceptive influence emanating from the locus coeruleus would explain why ipsilateral startle stimuli intensify the pain of CRPS (Knudsen et al., 2011). Alternatively, startle stimuli might trigger peripheral release of noradrenaline which then excites a nociceptive focus in the painful limb (Drummond and Finch, 2004). However, if this was the only mechanism, contralateral startle stimuli ought to have been as effective as ipsilateral startle stimuli in provoking pain (Knudsen et al., 2011). Moreover, we found that the pro-nociceptive influence of startle persisted in a subgroup of patients despite effective sympathetic blockade (Drummond and Finch, 2004). Thus, startle stimuli may also amplify the pain of CRPS by acting on disinhibited or sensitized central nociceptive circuits.

Hyperacusis (heightened sensitivity and discomfort to sound) is common in severely-affected patients with CRPS (de Klaver et al., 2007), particularly when acoustic stimuli are presented to the ipsilateral ear (Knudsen et al., 2011). Adrenergic brainstem nuclei provide both inhibitory and excitatory influences to the cochlear nucleus (Chikamori et al., 1980; Ebert, 1996), the first link in the neural pathway that underlies the acoustic startle response (Yeomans et al., 2002). Adrenergic and nociceptive signals also converge in the thalamus (Zhang et al., 1997) and limbic system (Ferry et al., 1997). Hence, a facilitatory adrenergic influence evoked by startle stimuli could contribute to hyperacusis in CRPS at multiple sites within the central nervous system.

Limb pain usually suppresses less intense pain elsewhere in the body, including the forehead (Knudsen and Drummond, 2009, 2011). Nevertheless, we found that sensitivity to pressure-pain *increased* in the forehead of CRPS patients during intensely painful stimulation of the affected limb, suggesting that inhibitory pain modulation processes had failed (Knudsen et al., 2011). This failure is consistent with a switch from anti-nociceptive to pro-nociceptive influences emanating from the locus coeruleus.

A shift toward pain facilitation in the rostral projections of the hemilateral pain modulation system might also explain the presence of hyperalgesia in the ipsilateral forehead of CRPS patients (Drummond and Finch, 2006; Finch et al., 2009; Knudsen et al., 2011). During attacks of migraine, allodynia extends within hours from the site of headache to the ipsilateral limbs (Burstein et al., 2000a,b), implying the spread of sensitization from primary nociceptive afferents and second-order neurons in the trigeminal dorsal horn to higher-order hemilateral convergent neurons. Similarly, sensitization might spread through pain circuits in CRPS, ultimately distorting sensory processing hemilaterally. Failure of hemilateral inhibitory pain control could accelerate this process (Figure 2). A similar explanation might account for nociceptive effects evoked by wearing prismatic goggles that deviate vision toward the affected side in CRPS (Sumitani et al., 2007), and by ambiguous visual stimuli (Cohen et al., 2012).

Some observations do not fit with the idea of a central adrenergic influence on pain in CRPS. For example, anecdotal reports suggest that tricyclic antidepressants help some patients (Rowbotham, 2006). Further trials are required to determine whether benefits can be identified in randomized controlled studies and, if so, whether this applies across the board or just to a subgroup of patients without central adrenergic mediation of pain. Whether benefits are due to decreased reuptake of noradrenaline in spinal pathways or some other mechanism (e.g., sodium channel blockade or down-regulation of spinal adrenoceptors or associated G-protein coupling) is also unknown.

## Future perspectives

Similar to most other chronic pain syndromes (Saariaho et al., 2011), a history of negative life events may increase vulnerability to CRPS (Geertzen et al., 1998). Although many processes could contribute to fear-pain conditioning (Bruijnzeel et al., 1999), from the current perspective it would be interesting to explore the involvement of brainstem adrenergic nuclei. For example, if the anti-nociceptive effects of central noradrenaline release transform into pro-nociceptive effects, activity in hyper-excitable fear and pain modulation circuits might lock in a cycle of chronic pain and distress.

In terms of treatment, benefits of α_1_-adrenoceptor blockade for CRPS have been described in case reports (Abram and Lightfoot, 1981; Stevens et al., 1993) but have yet to be evaluated in randomized-controlled trials. Nevertheless, involvement of α_1_-adrenoceptors at spinal or supraspinal levels is an intriguing possibility that may have potential treatment implications for certain patients with CRPS or other chronic neuropathic pain syndromes.

### Conflict of interest statement

The author declares that the research was conducted in the absence of any commercial or financial relationships that could be construed as a potential conflict of interest.
